# 

**Published:** 1781-01

**Authors:** 


LONDON:

Printed for the AUTHORS by W. RICHARDSON;

AND SOLD

By J. MURRAY, N° 32, Fleet-street.

MDCCLXXXI.

### DEATHS.

*Dec.* 26, 1780.—At his houſe in Harpur-ſtreet, London, aged 69, John Fothergill, M. D. F. R. & A. S. S. Member of the Royal Colleges of Phyſicians of London and Edinburgh, burgh, and of the Royal Medical Society at Paris; a phyſician eminently diſtinguiſhed for his learning and practical ſkill. His death was occaſioned by a ſuppreſſion of urine, owing to an enlargement of the proſtate. We are informed that Dr. Lettſom is engaged in writing his life, which he means to prefix to a complete collection of his works in 4to. ſo that we hope ſoon to be enabled to preſent our readers with ſome authentic memoirs of this juſtly celebrated phyſician.

*Jan.* 10, 1781.—At Bath, Mr. Philip Ditcher, ſurgeon.

19.—At his houſe in Union-court, Broad-ſtreet, London, aged 33 years, John Kooſtray, M. D. Member of the Royal College of Phyſicians, Secretary to the Medical Society, and Phyſician to the London Diſpenſary.

21.—At Kingſton, in Surry, after a lingering illneſs, William Lewis, M. B. F. R. S. and Member of the Royal Academy of Sciences at Stockholm.—We ſhall be thankful to any of our readers who will favour us with anecdotes of this ingenious phyſician.

### SECTION IV.

### MONTHLY CATALOGUE.

1. HEADS of Lectures on the Theory and Practice of Medicine. By *Andrew Duncan*, M. D. &c. &c. The second edition corrected and enlarged, 8vo. 427 pages. Edin. 1781.

Intended as a text-book for those who attend the author's lectures.

2. *Johannis Brunonis*, M. D. de Medicina Prælectoris, Societatis Regiæ Medicæ Edinensis Præsidis, Elementa Medicinæ. Small octavo, 421 pages. Edin. 1780.

3. Modern Improvements in the Practice of Physic, by *Henry Manning*, M. D. author of a treatise on the diseases of women. 8vo. 448 pages. London. 1780.

4. *Pauli de Czenpinsky*, Dissertatio Zoologico-Medica sistens totius Regni animalis Genera, in Classes, et ordines Linnæana Methodo digesta, præfixa cuilibet classi terminorum Applicatione. 8vo. Viennæ. 122 pages.

This is a useful work, especially for those who are beginners in the study of medical zoology.

zoology. The author ſometimes deviates from Linnæus in his arrangement. He has added the new ſpecies that have been diſcovered ſince the laſt edition of the *Syſtema Naturæ*. We are ſorry the additions are the leaſt frequent among the Mammalia. The work would have been leſs exceptionable if the author had omitted an idle ſtory related by Geſſner, from hearſay, of two nightingales converſing together in Latin. This tale did well enough in the laſt century. It would have been better likewiſe if the writer, inſtead of quoting ſcraps of morality from Auſonius, Cicero, and St. Auguſtine, had given us the real eſſential characters that diſtinguiſh the ſeveral ſpecies of *homo* from other animals.

5. Magazin für liebaber der Entomologie, i. e. a *Magazine for Lovers of Entomology*. By *J. C. Fueſsli*. 8vo. Zürich, with copper-plates.

An agreeable and uſeful work for all thoſe who employ themſelves on this branch of natural hiſtory.

6. *J. A. Scopoli*, Fundamenta Chemiæ Prælectionibus publicis Accommodata. 8vo. Pragæ. 166 pages.

The firſt part of this work treats of the objects jects and inſtruments of chemiſtry; the ſecond of its productions, and theſe are arranged and deſcribed according to the operations, as, 1ſt, calcination; 2d, reduction; 3d, ſolution; 4th, precipitation; 5th, diſtillation; 6th, ſublimation; 7th, mixture. The author every where adds the theory, but upon the whole we think his work far inferior to many other elementary books that have appeared on the ſubject.

7. *J. J. Plenk*, Chirurgiæ et artis Obſtetriciæ Profeſſoris Budenſis, Doctrina de Morbis Venereis. 8vo. 176 pages. Vienna, 1779.

This is a conciſe and judicious compilation of every thing that has appeared on the ſubject, occaſionally interſperſed with the author's own remarks, which in general are well founded.

8. Conamen Mappæ generalis Medicamentorum ſimplicium, ſecundum Affinitates Virium Naturalium, nova Methodo geographica diſpoſitorum, Auctore *Geo. Chriſtoph. Wurtz*, M. D. cum Tabula æenea. Argentor. 8vo. 221 pages.

This performance, which may appear whimſical to ſome readers, ſeems to be executed with accuracy, and muſt have coſt the author much pains. The map is 21 inches in length and 20 in width, and is neatly engraved.

9. Traité

9. Traité des Remedes Domeſtiques, pour faire ſuite au traité de la petite verole; par *M. Groſſin Duhaume*, Doĉteur Regent, ancien Profeſſeur des Inſtituts de Médecine en l'Univerſité de Paris, et Medecin de l' Hotel Dieu, Paris. 12mo. 172 pages.

10. Diſſertation contre l'uſage des bouillons de viande dans les maladies febriles; Par M. Paul Charles de Laudun, M. D. Medécin à Taraſcon en Provence. Paris. 12mo. 263 pages.

The reader will find nothing new or intereſting in this diſſertation. It is written to prove what is well known to every man of obſervation, that in a ſtate of fever, the ſick are totally averſe to fleſh broths, while they reliſh acidulated drink.

11. Conjeĉtures ſur le Temps où ont vécu pluſieurs anciens Medecins; par un Membre de la Société Patriotique de Heſſe Hombourg. Paris. 12mo. 83 pages.

This little work, which is written by M. Goulin, is intended to reĉtify ſome miſtakes into which he confeſſes to have fallen in his *Memoires Litteraires*, with regard to the time at which Aſclepiades flouriſhed.

12. In-

12. Inſtrucion curativa de las viruelas; por el *Don Joſef Amar*, M. D. &c. i. e. Remarks on the Cure of the Small Pox, by Don *Joſeph Amar*, M. D. phyſician to his Majeſty, &c. Madrid, 4to. 164 pages.

This performance will not impreſs the reader with a very favourable idea of the ſtate of phyſic in Spain. Don Amar begins his work with a hiſtory of the Small Pox, which is little more than a commentary on Rhazes. He declares himſelf an enemy to inoculation.

13. Lettre à M. de Brambilla, Ecuyer, premier Chirurgien de L. L. M. M. I. R. A. et de leurs Armées; par *M. de Lambon*, Ecuyer, premier Chirurgien de feu S. A. R. la Ducheſſe de Lorraine et de Bar, &c. &c. ſur trois Operations de la Symphyſe. Mons. 8vo. 25 pages.

The author of this letter has performed the ſection of the ſymphyſis pubis on two patients with ſucceſs. One of the two women has undergone this operation twice, and is now ſaid to be in perfect health, as well as the child that was brought into the world by means of the ſecond operation.

